# Beyond the Skin Plaques: Psoriasis and Its Cardiovascular Comorbidities

**DOI:** 10.7759/cureus.19679

**Published:** 2021-11-17

**Authors:** Chandra L Kakarala, Mohammad Hassan, Rishab Belavadi, Sri Vallabh Reddy Gudigopuram, Ciri C Raguthu, Harini Gajjela, Iljena Kela, Ibrahim Sange

**Affiliations:** 1 Internal Medicine, Jawaharlal Institute of Postgraduate Medical Education and Research (JIPMER), Pondicherry, IND; 2 Internal Medicine, Mohi-ud-Din Islamic Medical College, Mirpur, PAK; 3 Surgery, Jawaharlal Institute of Postgraduate Medical Education and Research (JIPMER), Pondicherry, IND; 4 Research, Our Lady of Fatima University College of Medicine, Hyderabad, IND; 5 Research, Tianjin Medical University, Tianjin, CHN; 6 Research, Our Lady of Fatima University College of Medicine, Valenzuela, PHL; 7 Family Medicine, Jagiellonian University Medical College, Krakow, POL; 8 Research, California Institute of Behavioral Neurosciences and Psychology, Fairfield, USA; 9 Research, K. J. Somaiya Medical College, Mumbai, IND

**Keywords:** anti-tumor-necrosis factor-alpha, anti-il12/23, systemic inflammation, cardiovascular risk, cardiovascular disease, psoriasis

## Abstract

Psoriasis, a widely prevalent chronic disease of the skin and joints, has long been associated with far-reaching systemic ramifications and decreased quality of life. However, psoriasis is largely underdiagnosed and insufficiently treated. Classical risk factors predisposing to cardiovascular diseases, such as hypertension, diabetes, metabolic syndrome, and dyslipidemia, have been noted in patients with mild and severe psoriasis. Furthermore, the magnitude of the cardiovascular comorbidity and the need to screen for risk factors has often been ignored while considering the management options for psoriasis. This article has reviewed the cardiovascular implications of psoriasis from the shared pathogenesis behind these two diseases to the increased incidence of cardiovascular events, such as myocardial infarction, stroke, and other causes of vascular mortality. Additionally, the therapeutic targets of common inflammatory pathways, such as those involving tumor necrosis factor α (TNF-α), interleukin-12/interleukin-23 (IL-12/IL-23), and helper T cells 17 (Th17), have been discussed with an emphasis on their efficacy in controlling psoriasis and its cardiovascular consequences.

## Introduction and background

Among the most prevalent immune-mediated disorders chronically involving the skin and joints, psoriasis manifests as symmetrical erythematous plaques with scaling [1]. Previously considered a similar entity to leprosy, it is now thought to affect at least 2% of the world's population, 30% of whom are expected to develop psoriatic arthritis later on [2-4]. Although psoriasis is seen to encompass a multitude of age groups, it is higher in adults (0.91%-8.5%) as compared to children (0%-2.1%) with a bimodal age pattern, wherein incidence peaks at 30-39 years and 60 years of age [5].

Developed countries with older populations showed an increased incidence relative to developing countries [6]. About 19 different genetic loci, including psoriasis susceptibility locus one (PSORS1) and other undesignated loci, have been implicated in psoriasis [7-9]. Environmental triggers such as streptococcal infection, smoking, trauma, and stress hasten the disease development in genetically predisposed individuals [9,10]. Psoriasis of the skin shows several clinical variants - plaque psoriasis or psoriasis vulgaris being the most common [11]. The sustained inflammatory pathways responsible for these dermatological manifestations are due to discrepancies in the innate and adaptive immune responses [5,12]. Keratinocytes and their interactions with dendritic cells, cytokines, monocytes, and other dermal cell types are responsible for the psoriatic plaque, namely the TNFα-IL-23-Th17 inflammatory pathway [11]. Autoantigens have also been well studied in the pathogenesis of psoriasis, such as cathelicidin (LL37), which strongly correlates to disease activity [13].

Psoriasis classically presents as well-circumscribed red papules with a grayish, dry scale on scalp, elbows, knees, lumbosacral region, or at the sites of trauma [1]. Diagnosis is clinical, and despite no permanent cure, treatment options for psoriasis include topical corticosteroid, calcipotriol, or combination therapy for mild to moderate disease [14]. Severe cases necessitate ultraviolet phototherapy, synthetic retinoids, methotrexate, and biologicals such as adalimumab [14]. Having been acknowledged by many as a systemic disease with far-reaching manifestations now, it is regrettable that psoriasis is a vastly underdiagnosed and untreated disease [14]. Despite customarily not affecting survival, psoriasis results in a significant de-escalation in the quality of life [15]. The psychological, metabolic, cardiovascular, and arthritic comorbidities are recently recognized as vital parameters to be considered during the management of psoriasis [16].

A preponderance of traditional cardiovascular risk factors is seen in patients with psoriasis, such as hypertension, diabetes mellitus type 2, obesity, metabolic syndrome, and dyslipidemia, the risk of which increases with greater psoriasis severity [17,18]. Common inflammatory pathways, cellular mediators, and genetic susceptibility are thought to be the unifying underlying mechanisms between psoriasis and cardiovascular disease [16]. This relationship should be explored further as treatment aimed at reducing inflammation or treatment involving biologicals may prevent major cardiovascular events, improve health goals and quality of life, and avoid permanent disability [16]. This review targets to explore the relationship between psoriasis and cardiovascular disease and the spectrum of cardiovascular events that the psoriasis-affected may be predisposed to and highlights the management options geared toward effective management of both psoriasis and its cardiovascular comorbidities.

## Review

Cardiovascular disease and psoriasis: shared pathogenesis

Although some extent of shared genetics contributes to the undeniable association of cardiovascular disease and psoriasis, this interrelation cannot be explained solely by genetic overlap, suggesting an alternative pathway that is basal to these two conditions [19]. The interaction of keratinocytes with various cell types and immune cells in the dermis leads to the development of the characteristic plaque seen in psoriasis [11]. This development consists of two distinct phases, an initiation phase that follows trauma, infection, or drugs, followed by a chronic clinical progression that makes up the maintenance phase [5,11]. Activation of dermal dendritic cells is the stimulus that triggers pathways leading to plaque formation [11]. Dendritic cells cause keratinocyte inflammation by stimulating Th17 cells by secreting IL-23 and proliferation of helper T cells one (Th1) by secreting interleukin (IL)-12 [11]. However, of paramount relevance is the fact that psoriasis is far from an isolated cutaneous inflammation and represents a chronic systemic inflammatory disease [19]. Several underlying pathogenic mechanisms shared by psoriatic and atherosclerotic plaque formation have been elucidated, one of the first being altered endothelial function and T-lymphocyte recruitment [19,20]. 

All the subtypes of T-lymphocytes involved in the development of psoriasis have been shown to be actively involved in atherosclerosis, especially Th1 and Th17 cells [21-23]. Th1 cells potentiate inflammation in keratinocytes by activating various mediators, such as interferon-gamma (IFN-γ), TNF-α, and IL-2 [22]. They are just as critical in atherosclerosis as it is thought to be driven by the trademark cytokine of Th1 activity, IFN-γ [24]. A factor increasingly seen to have a considerable impact on both these diseases is IL-17, a cytokine released by Th17 cells [19]. This is demonstrable by the finding that both patients with psoriasis and patients with acute coronary syndrome have increased circulating levels of Th17 cells [23,25]. Additionally, high levels of IL-17A messenger ribonucleic acid (mRNA) and protein have been documented in psoriatic plaques [23]. In keratinocytes, IL-17A induces severe psoriasis-like skin inflammation, and elsewhere, it triggers endothelial dysfunction, raises systolic blood pressure, and leads to hypertrophy of left ventricular musculature [26]. Further corroborating evidence about the involvement of IL-17 is evinced by the amelioration of skin lesions and partial vascular improvement on blockage of TNF- α and IL-6, which act downstream of IL-17A [26]. Regulatory T cells (Treg), whose primary role is to inhibit T-cell activation and proliferation, are also involved [27]. Through endothelial cell modulation, plaque stabilization has an anti-inflammatory role in atherosclerosis by decreasing macrophages and inhibiting pro-inflammatory cytokines [27]. In psoriasis, circulating and lesional Treg cells in the skin have impaired functioning [28]. Other mediators and cells are starting to be discovered, including neutrophils and their proteins, which have been shown to predict endothelial dysfunction and contribute to atherosclerosis development in psoriasis [29].

Neutrophils, which are widely seen in pustular and detectable in plaque-type psoriasis, are also shown to be heavy contributors due to their formation of Munro's abscesses in the epidermis [19]. In the vessels where they act on damaged endothelium, they release chemotactic agents to recruit leukocytes and increase foam cell development, a macrophage subset crucial to atherosclerosis [30]. The formation of low-density granulocytes (LDGs), a subtype of neutrophils that are known for their increased frequency of neutrophil extracellular traps (NETs), is recently being investigated in the pathophysiology of psoriasis and cardiovascular disease [29]. Macrophages, another part of the innate immunity system, are considered the hallmark of atherosclerotic changes in vessels, where they promote atherosclerosis progression by enhancing plaque necrosis and thinning of the protective fibrous cap and have been readily demonstrable in psoriasis as well [19]. Insulin resistance is a known stimulus for atherosclerosis, which is also a crucial pathogenic connection between psoriasis and cardiovascular comorbidity [31]. Chronic inflammation in psoriasis patients via cytokines induces insulin resistance in endothelial cells [32]. This, in turn, causes endothelial dysfunction and vascular stiffness once again as well as brings down the nitric oxide production, thus impairing the blood flow [32]. Adipokines, a family of mediators produced by adipocytes, were found to be in near-identical levels among the psoriasis patients and pre-diabetics, strongly suggesting a state of insulin resistance [31]. 

**Figure 1:**
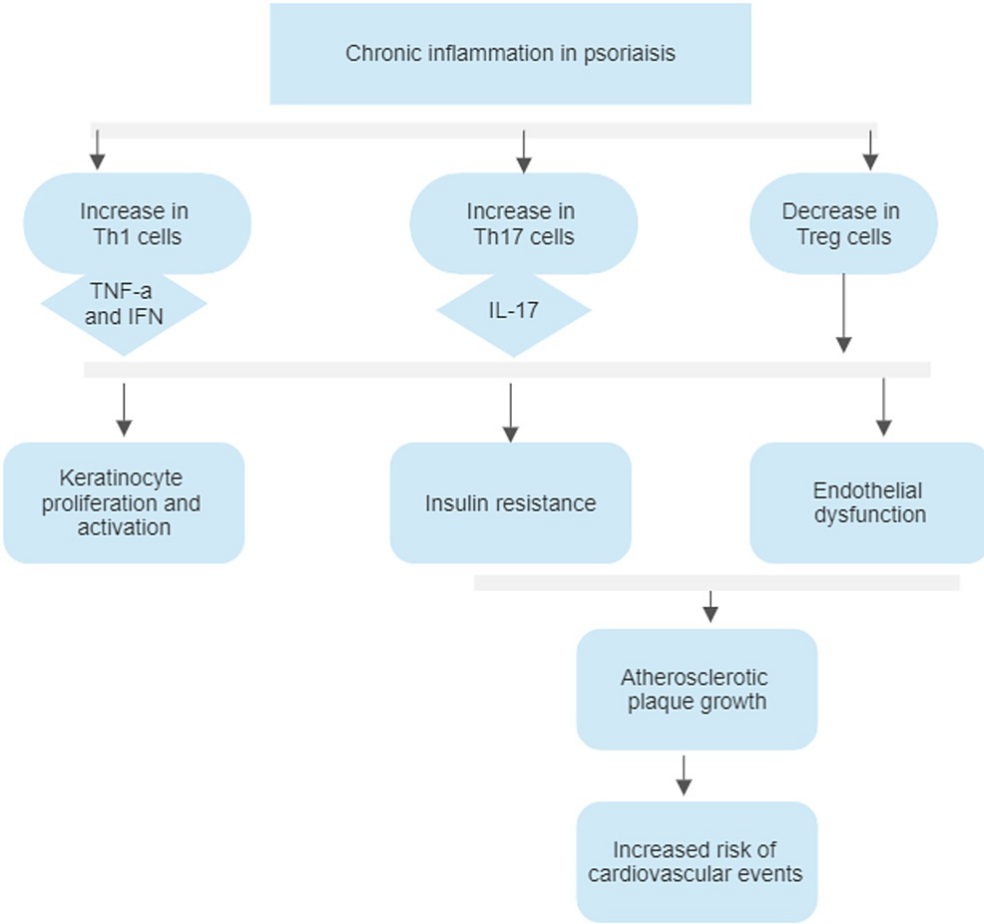
Summary of chronic inflammation in psoriasis leading to an increased risk of cardiovascular disease Th1 cells, Helper T cells one; TNF-a, tumor necrosis factor-a; IFN, interferon; Th17 cells, helper T cells 17; IL-17, interleukin-17; Treg cells, regulatory T cells.

Other pathogenetic pathways between psoriasis and the development of cardiovascular disease include a common state of vitamin D deficiency and oxidative stress [33,34]. While active vitamin D levels were reciprocally associated with psoriasis severity and Psoriasis Area and Severity Index (PASI), vitamin D has also been shown to be a powerful anti-inflammatory molecule, with protective results against cardiovascular disease [33]. A study by Playford et al. showed that low active vitamin D levels were associated with coronary plaque burden, visceral adipose volume, and increased cardiometabolic risk [33]. They also showed an inverse dose-response correlation of psoriasis severity and active vitamin D levels and an increase in those levels after a year of psoriasis treatment, thus establishing another strong pathogenetic link connecting diseases with low vitamin D, such as psoriasis, to the development of cardiovascular disease [33]. Similarly, oxidative stress is fundamental to the development of both diseases due to the release of pro-angiogenic factors, such as IL-8 and vascular endothelial growth factor (VEGF), which further cause increased leukocyte action and permeability at the areas of inflammation [34]. Reactive oxygen species from a similar enzymatic origin also determine the signaling pathways that contribute to plaque formation in atherosclerosis and psoriasis [34]. The summary of how chronic inflammation in psoriasis leads to an increased risk of cardiovascular disease development is shown in Figure 1.

Cardiovascular risk and events in psoriasis

The relationship between cardiovascular disease and psoriasis has been intriguing researchers for over five decades [35]. In 1973, Zweieten et al. were one of the first to prove an increased risk of occlusive vascular diseases, such as myocardial infarction, cerebrovascular accidents, and pulmonary embolization in the psoriatic patients compared to the non-psoriatic dermatological patients [35]. More recent studies have shown increased prevalence rates of typical cardiovascular risk factors in patients with psoriasis, such as hypertension, diabetes mellitus, dyslipidemia, obesity, and metabolic syndrome [36].

Risk Factors

As per the new American College of Cardiology/American Heart Association (ACC/AHA) guidelines, hypertension is said to be diagnosed if two or more blood pressure recordings are found to be ≥130 mm Hg systolic or ≥80 mm Hg diastolic [37]. Hypertension is one of the oldest associated risk factors with cardiovascular disease in a study by Rapsomaniki et al. showing that 66.6% of all adults who are hypertensive at 30 years have a 40% increased risk of developing a cardiovascular event compared to controls of similar age and sex [38]. They also develop major adverse cardiovascular events (MACE) at an earlier age, nearly five years before normotensive individuals [38]. A case-control study by Cohen et al. carried out in over 12,000 psoriasis patients found a significantly greater prevalence of hypertension in psoriasis patients (38.8%) than in controls (29.1%), after controlling other factors such as age, sex, smoking status, diabetes, and obesity [39]. Another study in female nurses in the United States also yielded a positive association between hypertension and psoriasis [40]. This study found the relative risk of developing hypertension to be 1.17 in patients with psoriasis compared to controls [40]. Refractory hypertension was also seen in greater frequency among psoriasis patients [41]. 

Diabetes mellitus is classically said to be a group of disorders characterized by fasting glucose levels of more than 126 mg/dL [42]. Hemoglobin A1c (HbA1c) ≥ 6.5% is another criterion used to diagnose diabetes mellitus [42]. Type two diabetes mellitus (T2DM) can be either a predominantly insulin-resistant state or a major secretory defect with peripheral insulin resistance and is commonly associated with metabolic syndrome and other diseases [42]. T2DM is also a well-known risk factor for cardiovascular disease, with the Framingham Heart Study showing a two- to four-fold increased risk of diseases such as myocardial infarction, stroke, peripheral arterial disease, and congestive heart failure in patients with diabetes [43,44]. Diabetes, too, has been documented to be strongly linked to psoriasis [45]. In a cohort study by Lee et al. in Taiwan, diabetes was found to be independently associated with a hazard ratio of 1.28 in mild psoriasis and 2.06 in severe psoriasis, after adjusting relevant comorbidities and medications [45]. Although a reliable estimate of diabetes in psoriasis patients is variable, it is unequivocally higher than that in the general population [41]. Several other studies have confirmed that psoriasis is an independent risk factor in developing diabetes mellitus in a dose-dependent form [46]. A population-based cohort study by Azfar et al. additionally found that psoriasis patients who develop diabetes are more likely to require systemic diabetic therapy [46]. 

Atherogenic dyslipidemia, distinguished by elevated plasma triglycerides, decreased high-density lipoprotein cholesterol and high levels of small low-density lipoprotein particles and has long been associated with cardiovascular disease [47,48]. It is seen in 35% of individuals with T2DM and 40% with metabolic syndrome [47,48]. Along with these risk factors, or alone, it can lead to atherosclerosis and, consequently, cardiovascular diseases, the incidence of which has been proven to be minimized after the reduction of cholesterol levels [44]. Dyslipidemia has also been observed in a greater frequency in psoriasis patients [49]. Compared to healthy controls, the mean levels of atherogenic lipids were found to be higher in psoriatic patients, while the antioxidant enzyme activities were significantly lower [49].

Obesity, which has been documented to double in prevalence since 1980 in more than 70 countries as per the Global Burden of Disease Group, is defined as a body mass index (BMI) of more than 30 and is commonly correlated with insulin resistance [50]. Obesity, due to increased insulin resistance, systemic inflammation, prothrombotic state, dyslipidemia, and endothelial dysfunction, has been shown to be associated with cardiovascular mortality, with the least mortality rates being in BMI ranges of 20-25 kg/m^2^ [44]. It has long been associated with psoriasis, with growing frequency as the psoriasis severity worsened [51]. In a population-based cross-sectional study conducted by Neimann et al. in the United Kingdom consisting of three groups (controls, patients with mild psoriasis, and patients with severe psoriasis), obesity was seen in 13.2%, 15.8%, and 20.7%, respectively [51]. 

The correlation of psoriasis with cardiovascular disease is also influenced by environmental and behavioral patterns, such as smoking, which is consistently associated with several inflammatory immune-mediated diseases [52]. The association of smoking and cardiovascular disease can be explained by different pathophysiological mechanisms such as oxidative stress, vascular influences, and interaction with signaling pathways [52]. Smokers have double the 10-year risk of MACE as compared to non-smokers, and passive smoking also contributes to an increased risk of cardiovascular disease [44]. Patients with psoriasis were found more likely to be active smokers, thus signifying another vital risk factor for cardiovascular disease [41]. In Germany, a retrospective study by Gerdes et al. found the prevalence of smoking and alcohol intake in a group of severe psoriasis patients higher than that of the general population [53]. In addition, disease severity correlated with the degree of smoking in both genders and alcohol intake in females [53]. Smoking was also found to impact the clinical severity and response to treatment in many patients [52].

Risk factors such as central obesity, glucose intolerance and insulin resistance, hypertension, low levels of high-density lipoprotein (HDL), and hypertriglyceridemia can be grouped into an entity called metabolic syndrome [41]. The prevalence of metabolic syndrome was found to be much higher in psoriasis patients (39.3%) as compared to controls (17.1%) by a study conducted by Kothiwala et al. in India [54]. They also found a prominent trend of the increasing prevalence of metabolic syndrome, T2D, and hypertension as the psoriasis duration and severity progressed [54]. Finally, the same study also showed significantly higher carotid intima-media thickness in patients with psoriasis (0.61 mm ± 0.01 mm) as compared to controls (0.37 mm ± 0.01 mm), suggesting a higher prevalence of subclinical atherosclerosis in psoriasis patients [54]. These significant associations of psoriasis with hypertension, diabetes, dyslipidemia, metabolic syndrome, obesity, and smoking are shown in Table 1.

**Table 1:** Summary of studies showing the prevalence of cardiovascular risk factors in psoriasis patients

References	Year	Design	Cases	Control	Population	Variable	Findings
Cohen et al. [39]	2010	Case-control study	12,502	24,285	Adults over 20, Israel	Hypertension	Prevalence in psoriasis patients - 38.8%, controls - 29.1%
Lee et al. [45]	2014	Cohort study	14,158	14,158	Adults, Taiwan	Diabetes	Associated with a hazard ratio of 1.28 in mild psoriasis and 2.06 in severe psoriasis
Neimann et al. [51]	2006	Population-based cross-sectional study	Severe psoriasis - 3854, mild psoriasis - 127,706		United Kingdom	Obesity	Prevalence in severe psoriasis - 20.7%, in mild psoriasis - 15.8%, and in controls - 13.2%
Kothiwala et al. [54]	2016	Cross-sectional study	140	140	India	Metabolic syndrome	Prevalence in psoriasis patients and controls - 39.3% and 17.1%, respectively

As an essential clinical consequence, the association of psoriasis with these comorbidities demonstrates the absolute need to regularly screen psoriasis patients for traditional cardiovascular risk factors and promptly commence treatment according to local guidelines [55].

Cardiovascular Events

MACE is a commonly used composite endpoint to describe cardiovascular severity [56]. Although a standard definition for MACE does not exist, it is generally said to include myocardial infarction (MI), stroke, and mortality from cardiovascular disease [56]. Additionally, it may consist of bleeding complications, cardiac arrest, revascularization, or rehospitalization for heart failure [56]. Independent of the cardiovascular risk factors they are predisposed to, patients with psoriasis have also been found to have an increased risk of MACE [57]. A cohort study by Mehta et al. found that, after adjusting for age, gender, diabetes, hypertension, tobacco usage, and hyperlipidemia, severe psoriasis was found to confer an additional 6.2% absolute risk of 10-year MACE [57].

According to its Fourth Universal Definition, acute MI has been defined as at least one value of troponin elevated > 99th percentile upper reference limit along with clinical features of myocardial ischemia, electrocardiogram changes suggestive of MI, or a new regional abnormal wall motion [58]. Acute MI remains one of the most common medical emergencies, with over eight million Americans presenting to the hospital annually with symptoms suggestive of MI [59]. After adjusting other risk factors, Gelfand et al. carried out a cohort study in the United Kingdom to eventually find an elevated relative risk of MI in patients with severe psoriasis compared to patients with milder versions [60]. This study showed incidences of 3.58, 4.04, and 5.13 per 1000 person-years for controls and patients with mild and severe psoriasis, respectively [60]. Additionally, the relative risk was seen to vary with age, the strongest association being in younger patients [60].

Stable coronary artery disease, identified by episodes of transient chest pain or angina following exercise, emotion, or stress, is one of the most important reasons for cardiovascular morbidity and mortality worldwide [61]. It is triggered by a reversible mismatch between myocardial blood supply and required oxygen, resulting in ischemia of myocardial tissue [61]. The mortality associated with coronary artery disease is as grave as to account for over one-third of the total deaths of both men and women in westernized countries [62]. Masson et al. uncovered an association between psoriasis and coronary artery disease similar to its interrelations with other cardiovascular diseases [63]. Through a cross-sectional study, they concluded that, compared to controls, the prevalence of coronary artery disease was higher in psoriasis patients (3.06% and 4.98%) [63].

Only recently have studies started to delve into the severity of the cardiovascular disease that psoriasis patients are prone to [64]. A cohort study of the Danish population by Ahlehoff et al. followed up psoriasis patients and controls after a first-time MI and revealed a significantly worse prognosis in psoriasis patients [64]. The incidence rates for all-cause mortality per 1000 patient-years was 119.4 in controls and 138.3 in psoriasis patients [64].

Stroke, or cerebrovascular disease, can be due to ischemic or hemorrhagic etiology and is commonly described as focal neurological symptoms developing acutely [62]. Approximately 700,000 new or recurrent strokes occur annually in the United States, making it the predominant cause of long-term disability [62]. Peripheral vascular disease, which frequently presents with intermittent claudication, is also due to systemic atherosclerosis, leading to critical narrowing of distal arteries [65]. In severe cases, it may also present with acute limb ischemia [65]. Both of these diseases are also seen in increased frequency in psoriasis patients [66]. After controlling for variables, a case-control study by Prodanovich et al. found the odds ratio of cerebrovascular disease in these patients to be 1.78 as compared to controls [66]. An even higher odds ratio (1.98) was seen on comparing the prevalence of the peripheral vascular disease among these two groups [66]. This study concluded that psoriasis itself is an independent risk factor for mortality as a higher percentage of deaths was found in patients with psoriasis (19.6%) when compared to patients without psoriasis (9.9%) [66].

Clinical research has shown that psoriasis is associated with an increased risk of cardiovascular events, like stroke and MI, as well as accelerated atherosclerosis [29]. Due to the closely related mechanistic pathways between psoriasis and atherosclerosis, effective screening and targeted treatment for both diseases are to be aimed at [29]. The summary of studies evaluating vascular events and outcomes in patients with psoriasis is depicted in Table 2. 

**Table 2:** Summary of studies evaluating vascular events and outcomes in patients with psoriasis MI- myocardial infarction

References	Year	Design	Cases	Controls	Population	Variable	Findings
Gelfand et al. [60]	2006	Prospective population-based cohort study	Mild psoriasis - 127,139, severe psoriasis - 3837	556,995	Patients with psoriasis aged 20 to 90 years, United Kingdom	MI	Incidences of 3.58, 4.04, and 5.13 per 1000 person-years for controls, patients with mild and severe psoriasis
Masson et al. [63]	2013	Cross-sectional study	1286	2547	All psoriasis patients older than 18, Buenos Aires	Coronary artery disease prevalence	Higher in the psoriasis group (4.98%) compared to controls (3.06%)
Ahlehoff et al. [64]	2011	Cohort study	462	48,935	People who experienced first-time MI during the period 2002-2006, Denmark	All-cause mortality after first-time MI	119.4 in controls and 138.3 in psoriasis patients per 1000 patient-years
Prodanovich et al. [66]	2009	Case-control study	3236	2500	Veterans hospital, Miami	Cerebrovascular and peripheral vascular disease	Odds ratio of 1.78 and 1.98, respectively

Targeted therapies

Disease severity, comorbidities, and access to healthcare are some of the factors affecting treatment options in psoriasis [11]. While vitamin D analogs and corticosteroids are used for mild to moderate diseases, severe disease is managed by systemic treatment such as methotrexate, cyclosporin A, retinoids, phototherapy, and biologicals [14]. From the vast amount of evidence supporting the association of MACE and psoriasis, queries have been raised about the potential of cutaneous psoriasis treatment to prevent heart attacks and lessen the cardiovascular risk development in the afflicted patients [67]. Patients with moderate to severe psoriasis especially may have decreased risk of cardiovascular comorbidities due to systemic inflammation suppression [68]. 

Methotrexate, a folic acid antagonist that inhibits nucleic acid synthesis, has anti-inflammatory and immune-modulating effects [69]. Its efficacy is noted to be lower than biologicals, but it is still widely used in psoriasis treatment due to its cost-effectiveness [69]. Significant side effects include nausea, anorexia, fatigue and malaise, hematopoietic suppression, and liver toxicity [69]. Prodanovich et al. were one of the first to definitively show a decreased cardiovascular disease incidence in psoriasis patients treated with anti-inflammatory medication [70]. This retrospective study found that long-term low cumulative dose methotrexate reduced the vascular risk in around 7,000 patients with psoriasis [70]. Additionally, in a case-control study by Lan et al. in Taiwan, patients using low-dose methotrexate had a lower risk of cerebrovascular disease when compared to those who have not been prescribed methotrexate [71]. 

Vitamin A derivatives are also used in the therapy of psoriasis in the form of retinoids, which assist by inducing keratinocyte differentiation and reducing epidermal hyperplasia, thereby slowing cell reproduction [72]. In the same study by Lan et al., retinoid therapy showed no variability in the risk of cerebrovascular disease development [71]. Retinoids have been shown to be less effective in yet another cohort study by Tsai et al., who found that there was a lower incidence of adverse cardiovascular outcomes, ischemic stroke, ischemic heart disease, and total mortality with methotrexate when compared to retinoids [73]. From the calcineurin inhibitor family, the T cell-inhibiting drug cyclosporine is also used for psoriasis treatment [11]. However, no protective cardiovascular effect was seen with cyclosporine usage in a Danish cohort study by Ahlehoff et al. comprising patients with severe psoriasis [74].

Biologics include receptor fusion proteins and monoclonal antibodies that target specific inflammatory pathways crucial in plaque development, like the IL-23/Th17 axis and TNF-α-signaling [11]. TNF-α is targeted by the first-generation biologics, namely etanercept, infliximab, certolizumab, and adalimumab [75]. Although the exact extent of benefit due to these drugs is unclear, most studies assert that TNF-α inhibitors decrease the risk of MACE [76,77]. In a clinical study by Hjuler et al., treatment with infliximab, etanercept, and adalimumab was seen to result in reduced coronary artery disease progression in severe psoriasis patients [76]. The controls in this study had increased coronary calcification upon follow-up compared to the patients treated with TNF-α inhibitors [76]. The efficacy of TNF-α inhibitors has been contrasted favorably with topical or oral/phototherapy treatment in another study, which showed much lower MACE risk in patients treated with TNF-α inhibitors [77]. These drugs are not without risks, and side effects include increased infection risk, injection site reactions, reactivation of latent diseases, lymphoma, and worsening heart failure [78]. Other biologicals include those targeting T helper cell activity such as ustekinumab, an IL-23 inhibitor that reduces Th17 cell proliferation, and briakinumab that also inhibits IL-12, thus minimizing Th1 cell activity [67].

Meanwhile, secukinumab and ixekizumab, IL-17 inhibitors, actively diminish the effects of IL-17, a downstream cytokine released by Th17 cells [78]. These cytokine-specific biologic agents have not been thoroughly scrutinized regarding cardiovascular effects, with varying results from different studies [79]. Briakinumab has been associated with an increased risk of adverse effects and has been discouraged for psoriasis therapy, with one randomized controlled trial showing a much higher risk of MACE due to briakinumab when compared to placebo [79]. However, a prospective observational study by Elnabawi et al. found anti-IL-17 agents to have the most significant percent reduction of plaque burden among all types of biologicals as well as a prominent decrease in the necrotic core [80]. 

**Figure 2:**
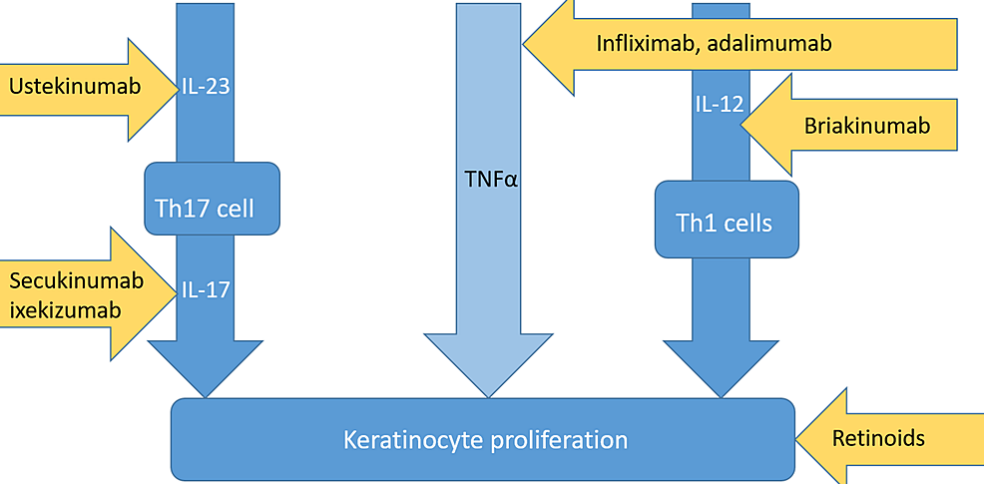
Summary of the different drugs against psoriasis and their mechanisms of action IL-23, Interleukin 23; Th17 cell, helper T cell 17; IL-17, interleukin 17; TNFα, tumor necrosis factor α; IL-12, interleukin 12; Th1 cells, helper T cells one.

Therapies used in conventional cardiovascular risk management should be used in caution with psoriasis patients, such as statins, aspirins, anti-hypertensive, and hypoglycemic drugs [81]. Although the pleiotropic actions of lipid-lowering statins include decreasing inflammation, different studies show both positive and negative effects of statins on psoriatic skin lesions [41]. From a prospective cohort study by Wu et al. to evaluate the effects of anti-hypertensive medication on psoriasis, beta-blockers were revealed to impart a higher risk of developing psoriasis [81]. Other anti-hypertensive drugs did not have an impact on psoriasis in this study [81]. In contrast, agents used in treating T2DM such as biguanides, thiazolidinediones, and glucagon-like peptide-1 receptor (GLP-1) agonists have been noted to be advantageous in combating psoriasis lesions as well [41]. Most importantly, risk factors for cardiovascular disease should be regularly screened, and lifestyle modifications like smoking cessation and weight loss should be implemented to prevent MACE in psoriasis patients [81]. An overview of the different sites that psoriasis drugs and biologicals act upon is shown in Figure 2.

Minimizing the cardiovascular toxicities of long-term systemic therapy such as acitretin, cyclosporine, methotrexate, and steroids is another vital part of reducing the incidence of MACE in psoriasis patients [82]. Acitretin has been recently shown to cause hyperlipidemia and can contribute to the development of coronary heart disease, while cyclosporine has long been associated with a dose-dependent increased risk of hypertension, hypercholesteremia, and hypertriglyceridemia [83,84]. Cyclosporine-related toxicities can be significantly reduced by regularly monitoring the cardiovascular effects as per well-defined protocols, by using it with other medication in the form of combination therapy, and by confining the total dosing time to under two years [82,84]. In contrast to these drugs, methotrexate has been shown to significantly improve the cardiovascular outcome in patients with psoriasis, lessening the vascular risk in a majority of studies [70,71]. Meanwhile, corticosteroids have been well-associated with metabolic side effects by altering lipid metabolism, causing hyperglycemia and blood pressure changes, thereby necessitating frequent metabolic profile monitoring [85]. A summary of the cardiovascular toxicities of different systemic drugs used in psoriasis is given in Table 3.

**Table 3:** Summary of the cardiovascular toxicities of different systemic drugs used in psoriasis

Drug	Cardiovascular Toxicity/Benefit
Acitretin	Hyperlipidemia, coronary heart disease
Cyclosporine	Hypertension, hypercholesteremia, and hypertriglyceridemia
Methotrexate	Lessens vascular risk
Corticosteroids	Dyslipidemia, hyperglycemia, and hypertension

Limitations

This article does not cover the more recent treatment options for psoriasis and their cardiovascular risks or benefits due to insufficient data. Additionally, the genetics common to cardiovascular disease susceptibility and psoriasis have not been evaluated in risk development.

## Conclusions

From the studies mentioned in this article, it is clear that psoriasis is far from limited to the dermis and has a wide range of comorbidities, including cardiovascular effects. The closely related pathogenetic pathways between psoriasis and vascular events have been reviewed in this article. Furthermore, relative risks of MACE, such as MI and cerebrovascular disease, have been shown to be elevated in several studies reviewed in this article. Treatment modalities that are successful in both psoriasis and preventing vascular diseases have been discussed, such as methotrexate and some biologicals. In summary, the clinical implication of this review article is to view psoriasis as a systemic disease and to highlight the prevalence of cardiovascular risk factors in psoriasis patients. Due to the critically high mortality rates associated with psoriasis comorbidities, it is vital to implement required prophylactic measures to prevent major cardiovascular disease and disability in these patients. This includes regular screening in all psoriasis patients for risk factors such as hypertension, diabetes mellitus, metabolic syndrome, and dyslipidemia and initiating treatment for detected risk factors. As psoriasis is underdiagnosed and under-treated in several areas, this article emphasizes the necessity to manage psoriasis early so as to avoid cardiovascular morbidity later on. More comprehensive studies on biologicals and other treatment modalities must be undertaken to undercover the cardiovascular consequences of commonly used psoriatic medication. Additionally, if psoriasis patients will benefit from extra cardiovascular risk prevention measures compared to the general population remains to be seen.
